# Quality aspects relating to giving birth in Switzerland: An analysis of quality indicators in inpatient obstetrics from 2013 to 2017

**DOI:** 10.3389/fpubh.2022.1009412

**Published:** 2022-10-13

**Authors:** Dominik Moser

**Affiliations:** ^1^Department of Health Care Management, Institute for Technology and Management, Technical University Berlin, Berlin, Germany; ^2^Organization and Services Department, Operations, GZO Hospital Wetzikon/Zurich, Wetzikon, Switzerland; ^3^Economics and Technology Department, Swiss Distance University of Applied Sciences (FFHS), Brig, Switzerland; ^4^School of Medicine, University of St. Gallen, St. Gallen, Switzerland

**Keywords:** obstetrics, quality variation, quality transparency, hospital care planning, public reporting

## Abstract

Quality transparency supports the reduction of information asymmetries in the health care system and enables the targeted regulation of health care. This study examines quality variation in inpatient obstetric care using the official Federal Office of Public Health Inpatient Quality Indicators (CH-IQI; vaginal births with 3rd- and 4th-degree perineal tears, vaginal births with episiotomy, and Caesarean section for low-risk births). It includes 101 maternity hospitals and 425,810 births between 2013 and 2017. For births with perineal laceration of 3rd and 4th degree, Switzerland performs 0.9% poorer in comparison to Germany (D-IQI) and Austria (A-IQI). For births with episiotomy, Switzerland is 1.1% above Germany. The Caesarean section rate for low-risk births was 26.8% in Switzerland in 2017 (Germany: 25.9%). When comparing Swiss clinics, private clinic locations in particular stand out. One possible reason for this may be the density of care, patient demands or the system of affiliated physicians at these clinics.

## Highlights

- Inpatient quality indicators show variation among maternity hospitals in Switzerland.
- Vaginal births with 3rd/4th degree perineal laceration show low quality variance.
- Relevant quality differences exist in perineal incisions and Caesarean section rates.
- Results of six private clinics are clearly above the expected values.
- Country comparison with Germany and Austria shows potential for improvement in Switzerland.

## Introduction

The criteria for choosing a hospital are similar across various countries: previous hospital experience, geographical accessibility, recommendations from specialists and the personal environment, image, and infrastructure all influence the choice of hospital (1). In Switzerland, the hospital nearest to the place of residence can be selected based on the cantonal hospital list. Depending on the insurance model, a cross-cantonal choice of hospital is possible.

The proximity of a maternity hospital is often an emotive topic for hospital capacity planners and health politicians. In Switzerland, politicians will frequently face difficulties being elected if they dare to publicly criticize a small (maternity) hospital. There is hardly any medical specialty in Switzerland where future patients make such conscious decisions as concerning which maternity hospital to choose (2, 3). Among important factors are past experience of giving birth and recommendations from a gynaecologist or private reference persons, such as friends or family members (3). Furthermore, several marketing activities aid patient acquisition and provide direction to the patients, e.g., prenatal courses for parents, parent insight events or pregnancy internet-blogs. The selection of a maternity hospital is mostly based on structural criteria such as the distance of travel to the hospital, the availability of a neonatology, or whether family rooms or hotel services are offered. Process and outcome quality indicators are normally not taken into consideration.

Certifications such as the “Baby-Friendly Hospital” quality label from UNICEF and the WHO could help ensure process quality. ISO certification to demonstrate process-based and structured practice would also be conceivable (4). The quality of outcomes is annually analysed and published by the Federal Office of Public Health (FOPH) in Switzerland. These quality indicators for Swiss acute hospitals (CH-IQI; Swiss inpatient quality indicators) can be compared with those of Germany and Austria. Benchmarks are non-public. Hospital-specific evaluations are not published. There is hardly any public discussion about these results and benchmarks.

In Switzerland, the Federal Law on Health Insurance stipulates which services are paid for by mandatory health insurance (including pregnancy and maternity). All services must be effective, expedient, and economical. The effectiveness must be proven according to scientific methods (5). To ensure this standard of effectiveness, high patient safety and treatment quality, all three quality perspectives must be considered for hospital capacity planning purposes (6). The present study therefore addresses the following question: What is the variation in quality of the official quality indicators for inpatient obstetrics at acute care hospitals in Switzerland?

This study is intended to contribute to quality transparency and encourage discussion concerning quality in the health care system. The study does not contain any fundamental and medical questioning of the currently valid quality indicators. For the further development of quality indicators, a transparent and transnational process has been available for many years; it is published on the website of the FOPH.

## Materials and methods

In the study, the available quality data relating to inpatient births in Switzerland is used to present the quality variation descriptively. Due to a lack of structural data of individual medical specialties, explorative or causal investigations are unfortunately not possible (e.g., the staffing ratio influences, the quality of treatment). The sources of the study data in the first part are the quality indicators for Swiss acute hospitals (CH-IQI) in combination with the Swiss hospital structure data (7). Both data sets are from the Swiss Federal Office of Public Health (FOPH) and include all service providers in Switzerland, as they are based on a legal obligation to provide information to the government (8). Data delivery by hospitals to health and statistical authorities is required by law in Switzerland. CH-IQI is based on the hospitals' accounting data, which is why it can be assumed that the data quality is high. Following quality initiatives in the United States, IQI was adapted for Germany, Austria, and Switzerland (9). The first evaluations in Switzerland were conducted in 2008/09. As of that date, there has also been a transparent process for the further development of the quality indicators, in which professional medical societies and experts can participate. Data from Austria and Germany is used for comparison (10, 11).

The study includes 101 maternity hospitals in Switzerland, in which a total of 425,810 babies were born between 2013 and 2017. All maternity hospitals in Switzerland in which babies were born in each year of the study period are considered. Of all the maternity hospitals, 15 are specialized birthing centres, 1 is a clinic specialized in gynaecology, and 85 are general hospitals (primary, centre, and university providers). On average, 4,216 babies were born per location over the 5 years (median: 3,045; 24 births at the smallest hospital; 20,084 at the largest hospital).

In the following, the CH-IQI quality indicators, the proportion of vaginal births with 3rd and 4th degree perineal tear (G.1.2.P), the proportion of vaginal births with episiotomy (G.1.3.P) and the proportion of Caesarean section in low-risk birth (G.1.5.P) are considered in detail. The expected rate of all maternity hospitals is calculated by the FOPH, is the same nationally and corresponds to the Swiss average (according to the quality indicator specification 5.1) (8).

In a third-degree perineal tear, the sphincter muscle is partially or completely torn. Healing may take weeks to months. In a fourth-degree perineal tear, both the sphincter and the intestinal mucosa are torn; as a result, surgery may be necessary and healing may take several months. An episiotomy is a manual incision to assist in the birth process; in this case, healing may take weeks to months. Caesarean section in low-risk birth can be done at the patient's request, it can however also be carried out for economic reasons (a hospital earns more from a Caesarean section). In addition to the common risks of surgery (wound healing problems, pain, etc.), there is an increased risk that the baby will develop secondary problems such as respiratory problems, asthma, or diabetes (12). Currently, it is thought that this can be reduced, at least in part, by the microbiota, although the long-term consequences are still unclear. In addition, the costs for health insurance companies are higher.

The visualization of the results is carried out by means of a risk-adjusted plot (13). For the calculation, the standard method “Wald confidence interval” is used and is shown as a funnel plot (14). Due to the amount of data, it can be assumed that the risk-adjusted sample is normally distributed and corresponds to a Poisson distribution. Furthermore, it can be assumed that the mean of the sample (λ) is 100% and thus one can assume a lambda of 1.

## Results

Table 1 shows a country comparison of Switzerland in 2013–2017, Austria in 2017 and Germany in 2017; not all hospital stays are included in Germany (8, 10, 11). The values for all countries were aggregated and anonymized by the authorities. For Switzerland, the values are public if more than 30 births were reported for a hospital. The confidence interval should accordingly be interpreted with reservation, as low values are not considered. Based on the single values of one year for all countries, will not be any statistical tests for assessing the relevance of the differences (parametric or non-parametric). For vaginal births with perineal laceration of the 3rd and 4th degree (G.1.2.P), Switzerland shows a higher value of 3% (CI = 0.2920, 0.3356; year 2017: 2.9%) than Germany (2%) and Austria (2%). The quality indicator perineal laceration at birth (G.1.3.P) provides a similar picture: Switzerland (21.5%; CI = 0.18915, 0.22838; year 2017: 16.8%), Germany (15.3%). Austria does not report any data here. The rate of Caesarean section at low-risk birth (G.1.5.P) shows a less clear picture: Switzerland (27.8%; CI = 0.26870, 0.30469; year 2017: 26.8%), Germany (25.9%). Here, too, Austria provides no data or only the total Caesarean section rate of all births.

**Table 1:** Quality indicators of obstetrics in comparison between Switzerland, Austria and Germany.

	**Switzerland (CH-IQI)**	**Austria (A-IQI)**	**Germany (D-IQI)**
	**2013-2017**	**2017**	**2017**
G.1.2.P Proportion of vaginal births with perineal tears of 3rd and 4th degree	3% (0.2920, 0.3356)	2%	2%
	(8,500 cases)	(1,213 cases)	(10,405 cases^*^)
G.1.3.P Proportion of vaginal births with episiotomy	21.5% (0.18915, 0.22838)	-	15.3%
	(60,762 cases)		(80,070 cases^*^)
G.1.5.P Proportion of Caesarean section in low-risk births	27.8% (0.26870, 0.30469)	-	25.9%
	(103,282 cases)		(172,264 cases^*^)

* Germany: data collection not complete.

**Figure 1:**
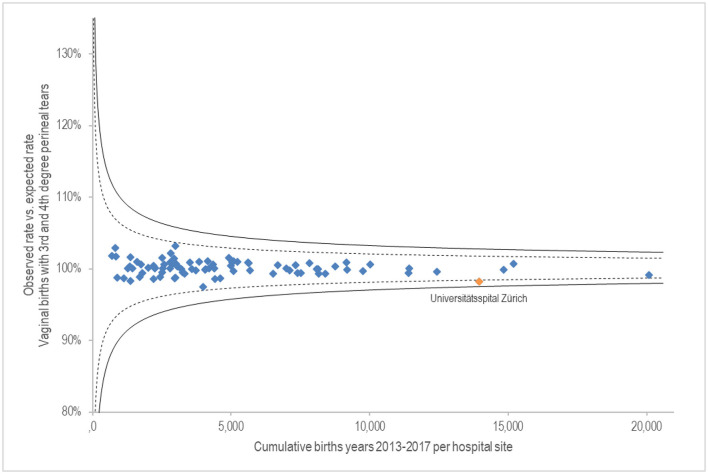
G.1.2.P Proportion of vaginal births with perineal laceration of 3rd and 4th degree.

The x-axis of Figure 1 shows cumulative births from 2013 to 2017 per hospital site. The y-axis shows the degree of fulfilment of the observed proportion of vaginal births with 3rd- and 4th-degree perineal tears, compared with the risk-adjusted expected rate. At 100%, the observed rate is equal to the expected rate. When above 100%, the observed Caesarean rate is higher than expected; below 100% means that fewer Caesareans than expected were performed. Due to the lack of plausibility, 17 sites with a rate of 0% (including 12 birth centres) were excluded. The result shows a small quality variance. Slightly positive is the University Hospital Zurich with 1.8% below the expected rate with a relatively high number of births of 13,948 over 5 years.

**Figure 2:**
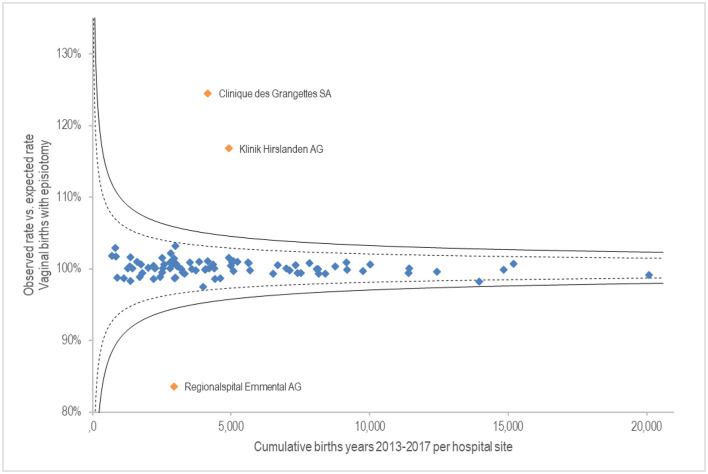
G.1.3.P Proportion of vaginal births with episiotomy.

Figure 2 shows on the x-axis the cumulative births from 2013 to 2017 per hospital site. The y-axis shows the fulfilment rate of the observed rate of vaginal births with episiotomy compared to the risk-averse expected rate. Twelve birth centres were excluded with a proportion of 0% because their results were untraceable (3,860 births). The results vary widely. Three sites deviate more than 10% from the standard deviation of 99%. Above the expected rate are two sites of the private hospital group Hirslanden (together 9,061 births over 5 years); clearly below the expected rate is the Emmental regional hospital (2,928 births).

**Figure 3:**
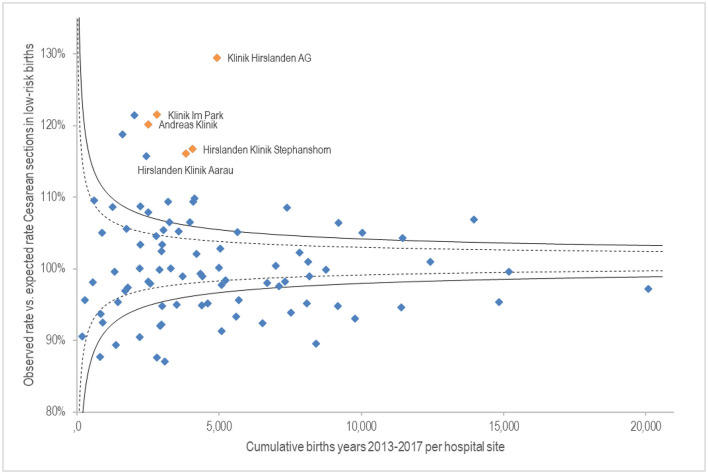
G.1.5.P Proportion of Caesarean section in low-risk births.

Figure 3 shows on the x-axis the cumulative births from 2013 to 2017 per hospital site. The y-axis shows the fulfilment level of the observed Caesarean section rate compared to the risk-averse expected rate for low-risk births. Sites with no Caesarean deliveries (15 birth centres) were excluded. Results show extensive quality variance. Labelled hospitals are those with variance starting at 10%, based on a standard deviation at 99%. All labelled hospital sites belong to the Hirslanden private hospital group and are significantly above the expected Caesarean rate.

## Discussion

Opinions diverge on what constitutes quality in pregnancy and obstetrics. The World Health Organization (WHO) published various guidelines for intrapartum care for a positive childbirth experience or maternal and newborn care for a positive postnatal experience (15, 16). The guidelines on birth contain 56 recommendations in three categories: Recommended, Not recommended, and Recommended only in specific contexts. At the second stage of labour, for example, the WHO describes that routine or liberal use of episiotomy is not recommended. For maternal and newborn care they recommend for example non-pharmacological interventions to prevent postpartum mastitis.

The professional medical societies for gynaecology and obstetrics in Switzerland, Germany and Austria jointly published comprehensive guidelines on birth, for example Caesarean section (Sectio caesarea) or vaginal birth at term (17, 18). They contain scientifically based guidelines on indications as well as treatment phases and techniques. As up to 10% of all births are terminated by vaginal surgery, guidelines are currently being developed. Vaginal-operative obstetrics is understood to mean birth by vacuum extraction, forceps or—in an extended sense—by the Kristeller manoeuvre (19).

The results show three of the mandatory quality indicators of the obstetrics specialty in Switzerland: vaginal births with perineal tear of 3rd and 4th degree, vaginal births with perineal tear and low risk Caesarean deliveries.

The results of vaginal births with perineal tear of 3rd and 4th degree provide an unremarkable picture, independent of the number of births. A stable quality with little variability can be assumed here. The University Hospital of Zurich stands out as minimally positive. Aasheim et al. (20) conclude in a meta-analysis that massage and warm compresses can reduce serious third and fourth degree perineal injuries. However, they also identify a need for further research into effective perineal protection techniques.

Two hospitals of the private hospital group Hirslanden are conspicuous in the proportion of vaginal births with episiotomy. From a medical perspective, an analysis of the situation by specialists would certainly be indicated. Conceivable factors include potential complications during birth, uncertainty of the treatment team, or productivity pressure on resources such as staff or delivery rooms (21). A routine episiotomy is described as obstetric violence by the WHO and numerous professional medical societies (22, 23). Likewise, research results show that there is a broad consensus that episiotomies should be considered a scientifically unfounded procedure and that the rate of episiotomies should be reduced accordingly. However, the criteria for when an episiotomy is indicated (selective episiotomy) are inconsistent. Frequently mentioned criteria for a selective episiotomy are primiparity, foetal weight of more than 4 kg, prolonged second stage, operative delivery and shoulder dystocia (22). A scientific consensus on the indication and the technique to be used is necessary to increase quality and create legal certainty for all parties involved. Likewise, from a medical point of view, special attention must be paid to consent and information, as this is often given verbally shortly before the intervention (23).

Finally, the proportion of Caesarean sections in low-risk births: five hospitals of the Hirslanden private hospital group are conspicuous here. This may be due to the desire of the patient, who often has additional insurance. An analysis of the patients with supplementary insurance would certainly be interesting. However, the data relating to this is not public. It is also conceivable, however, that this is because hospitals and general practitioners, who frequently run a private practice as a sideline, can plan more easily. In addition, Caesarean sections are more highly remunerated. The Emmental regional hospital is strikingly positive. The hospital is certified as a “Baby-Friendly Hospital” by UNICEF, which generally seeks to promote natural birth. In addition, it may also be due to its rural location, where people tend to prefer natural birth. The increase in elective section in the last two decades cannot be explained by an increase in high-risk pregnancies (24).

Compared to Austria and Germany, quality results must be assumed to be below average. One possible reason for this may be the structure of care, for example due to the number of affiliated physicians in Switzerland, or the density of care in hospitals and birth centres.

In addition to the current quality indicators, there are other common practices such as the Kristeller manoeuvre or fundal pressure that can influence the birth experience and quality. The Kristeller manoeuvre involves applying strong pressure to the upper part of the uterus (fundus uteri) during the expulsion phase. Malvasi et al. (25) note a large discrepancy between the use of the Kristeller manoeuvre or fundal pressure during labour vs. juridiction in Europe and the USA. The authors therefore recommend guidelines and recommendations on which manoeuvre techniques can be used and under what circumstances. Because fundal pressure is not beneficial to women and is potentially harmful, the WHO and other bodies strongly advise against it (26–28). Ferrington et al. (26) examined 76 studies from 22 countries and found that uterine fundal pressure is still widespread. More efforts are needed to prevent the potentially unnecessary and harmful management, they said. This is especially true as the possibility of the mother refusing the manoeuvre during childbirth is limited (27).

The study also has limitations and can be criticized. The data set is already several years old and covers the years 2013 to 2017. The calculation of the confidence interval can also be criticized. The “Wald confidence interval” is a common instrument, but in some cases more suitable instruments are described (14). Furthermore, the study does not contribute to a possible economic perspective. For example, revenues from Caesarean sections are higher than from natural births. However, the revenues vary depending on the hospital, as there are different base rates, which are multiplied by the coded case severity (case mix). In addition, there are supplements for patients with supplementary insurance. It can also be criticized that no medical causes are analysed; this is correct, since the focus of the study is on the descriptive presentation of already existing and accepted quality indicators. The study is intended to contribute to quality transparency and stimulate a quality discussion. The development and improvement of the individual quality indicators as well as the detailed case and cause analysis, are the responsibility of the specialists in their respective disciplines.

## Conclusion and outlook

The study contributes to the quality transparency of inpatient care and stimulates a quality discussion in the field of obstetrics. No major deviations can be found in vaginal births with 3rd and 4th degree perineal tears. On the other hand, abnormalities were found in vaginal births with perineal tears and in the proportion of Caesarean sections in low-risk births. One private hospital group in particular stands out here. It is worthwhile to reflect on the data with experts. In addition to reflection in professional circles, a public discussion on quality would also be desirable. In addition to the medical reasons, discrepancies could also be due to the needs of the patients; also conceivable are reasons of convenience with regard to planning as well as economic reasons (higher profits).

For the future, a continuous and transparent presentation and evaluation of the quality indicators would be desirable. These quality standards are to be further developed by the professional medical societies in terms of content and coordinated internationally. In addition, care must be taken to avoid potentially negative effects of quality transparency. Also conceivable would be certification standards around obstetrics and quality-oriented regulation in hospital care planning.

## Data availability statement

Publicly available datasets were analyzed in this study. This data can be found here: https://www.bag.admin.ch/bag/de/home/zahlen-und-statistiken/zahlen-fakten-zu-spitaelern/qualitaetsindikatoren-der-schweizer-akutspitaeler.html.

## Author contributions

The author confirms being the sole contributor of this work and has approved it for publication.

## Conflict of interest

DM was employed part-time in an acute somatic hospital with an obstetrics department in the canton of Zurich (Switzerland) and also be a visiting researcher at the School of Medicine (University St. Gallen, Prof. Alexander Geissler).

## Publisher's note

All claims expressed in this article are solely those of the authors and do not necessarily represent those of their affiliated organizations, or those of the publisher, the editors and the reviewers. Any product that may be evaluated in this article, or claim that may be made by its manufacturer, is not guaranteed or endorsed by the publisher.
